# Telehealth-enabled behavioral treatment for problem behaviors in boys with fragile X syndrome: a randomized controlled trial

**DOI:** 10.1186/s11689-020-09331-4

**Published:** 2020-11-20

**Authors:** Scott S. Hall, Katerina D. Monlux, Arlette Bujanda Rodriguez, Booil Jo, Joy S. Pollard

**Affiliations:** 1 Department of Psychiatry and Behavioral Sciences, Stanford University School of Medicine, Stanford, CA USA; 2 Behavior Change Institute, Oakland, CA USA

**Keywords:** Fragile X syndrome, Problem behavior, Functional analysis, Behavioral treatment, Randomized controlled trial

## Abstract

**Background:**

Children with fragile X syndrome (FXS) are at increased risk for exhibiting problem behaviors such as aggression and self-injury. However, many children with FXS have limited access to behavioral treatments that have known efficacy due to the low availability of treatment providers and the wide geographical dispersion of families with FXS across the country. Telehealth may offer a cost-effective and practical solution to overcome these significant barriers. We examined the effect of administering an established behavior analytic intervention called functional communication training (FCT) via telehealth on levels of problem behaviors exhibited by boys with FXS. We also examined treatment acceptability, as well as the effect of the treatment on levels of parenting stress.

**Methods:**

Boys with FXS, aged 3 to 10 years, who displayed problem behaviors daily, were randomized to receive FCT via telehealth (*n* = 30) or treatment as usual (*n* = 27) over 12 weeks. Outcome measures included in-session observations of problem behavior, the Aberrant Behavior Checklist—Community (ABC-C), the Treatment Acceptability Rating Form—Revised (TARF-R), and the Parenting Stress Index, 4th edition (PSI-4).

**Results:**

Intention-to-treat analyses indicated that scores on the irritability subscale of the ABC-C, our primary outcome measure, decreased significantly for boys who received FCT via telehealth compared to boys who received treatment as usual (*p* < .001, Cohen’s *d* = 0.65). In-session observations conducted for those who received treatment showed that levels of problem behavior decreased by 91% from baseline. Levels of parenting stress related to child behavioral problems were also lower following FCT treatment, and caregivers reported that the intervention was acceptable.

**Conclusions:**

These findings support telehealth-enabled FCT as a framework for expanding access to behavioral treatments for problem behaviors in children with FXS. Expanded delivery of behavior analytic treatment via telehealth also has the potential to lower healthcare costs, improve child and family quality of life, and lead to advances in the treatment of problem behavior in the broader population of individuals with neurodevelopmental disorders.

**Trial registration:**

ClinicalTrials.gov, NCT03510156. Registered 27 April 2018

## Background

A significant proportion of boys with fragile X syndrome (FXS), the leading known inherited cause of intellectual disability, commonly display high rates of problem behaviors such as aggression, self-injury, and property destruction that can be extremely distressing to the individual and families concerned [1–5]. Prevalence studies suggest that problem behaviors occur at significantly higher rates in FXS than would be expected for the general population of individuals with intellectual disabilities [6–8]. Children with FXS who exhibit problem behaviors may be at increased risk of restrictive physical interventions, placement in restrictive school or residential settings, and inhibited educational and social progress. In addition to the substantial financial and social costs, families who care for a child with FXS experience high levels of stress, as well as increased mental and physical health concerns [9–12]. There is, therefore, a critical need for the development of effective, acceptable, and sustainable treatments to ameliorate problem behaviors in FXS [13].

FXS occurs due to an expansion of > 200 CGG trinucleotide repeats in the promoter region of the fragile X mental retardation-1 (*FMR1*) gene at locus 27.3 on the long arm of the X chromosome affecting approximately 1 in 4000 males and 1 in 8000 females in the general population [14–16]. Methylation of the gene results in reduced or absent fragile X mental retardation protein (FMRP), a key protein involved in synaptic plasticity and dendritic maturation in the brain, with males being more affected than females [17, 18]. As a consequence, males with FXS exhibit a significant number of developmental and cognitive deficits [19], including impairments in executive functioning, visual memory and perception, social and communication skills, and increased risk for autistic-like behaviors [20–24]. A significant proportion of males with FXS are prescribed psychoactive medications (e.g., antipsychotics, stimulants) [25, 26] to manage behavioral symptoms. However, the outcomes of medication trials for FXS have been extremely variable, and there is currently no FDA-approved pharmacological treatment for FXS [27–29].

Although treatments for FXS are often considered from a medical perspective, several studies have shown that problem behaviors displayed by children with FXS may be shaped and maintained by operant learning processes such as positive and/or negative reinforcement [30–33]. For example, if a child typically receives attention from others when he/she engages in problem behavior, the child’s problem behavior may be positively reinforced by attention. Similarly, if demanding tasks and/or transitions are typically removed when the child engages in problem behavior, the child’s problem behavior may be negatively reinforced by escape from demanding activities. In other words, problem behaviors in FXS may serve different learned purposes or functions. Unless these functions are specifically addressed as part of the child’s treatment regimen, interventions that do not consider the function(s) of the child’s problem behavior are unlikely to be effective in the long term [13, 34].

One behavioral treatment that specifically addresses the function(s) of the child’s problem behavior is functional communication training (FCT) [35–37]. In this approach, the focus of treatment is to ensure that problem behavior no longer results in reinforcement while simultaneously teaching the child to engage in alternative, more appropriate forms of communicative behavior that serve the same function(s) [38, 39]. For example, if the function of a child’s problem behavior is to gain access to attention, the focus of treatment would be to ensure that the child no longer receives attention when problem behavior occurs, while simultaneously teaching the child an alternative communication strategy in order to gain access to attention more appropriately. Similarly, if the function of a child’s problem behavior is to escape from tasks, the focus of treatment would be to ensure that tasks are no longer removed when the problem behavior occurs while simultaneously teaching the child an alternative communication strategy in order to escape from the tasks more appropriately. This procedure contrasts with other behavioral interventions such as Parent-Child Interaction Therapy that do not consider the function(s) of the child’s problem behavior [40].

A significant barrier to conducting behavioral treatment research in children with FXS is the wide geographical dispersion of families across the country, with many families living in rural and medically underserved communities. Furthermore, the majority of clinics that specialize in FXS are located in larger cities leading to critical health access disparities for rural populations. Given that the World Health Organization has designated a family’s geographical location as the most significant barrier to behavioral treatment access [41], it is not surprising that many individuals with FXS are excluded from receiving access to behavioral treatment. Due to the shortage of specialized treatment centers, many families with FXS may also be required to travel long distances to access critical behavioral treatment. There are a limited number of qualified professionals available to render behavioral health services in many rural areas. Delivering behavioral treatments conventionally (i.e., in-person) may, therefore, not be feasible for many families with FXS. Telehealth, defined as the delivery of health-related services and information via telecommunication technologies, might be a practical and cost-effective solution to overcome these barriers. Telehealth is used throughout the healthcare industry [42–45] and is effective for training professionals to conduct a variety of behavioral interventions [46, 47]. The use of telehealth may therefore be critical to advancing behavioral therapeutic horizons for children with FXS and their families.

To date, a few small-scale studies have administered behavioral interventions via telehealth, and these interventions have focused on increasing adaptive behaviors in the child’s repertoire rather than specifically targeting problem behaviors [48, 49]. In a randomized controlled trial of a spoken language intervention for 20 young boys with FXS, for example, children who received the intervention in weekly sessions over 12 weeks were found to display increased use of verbal utterances during a story-telling activity compared to those who received treatment as usual [50, 51]. The feasibility of implementing the Early Start Denver Model via telehealth for two young boys with FXS has also been described [52]. However, to our knowledge, no randomized controlled studies have evaluated the efficacy and acceptability of delivering behavioral interventions via telehealth to decrease problem behaviors exhibited by children with FXS.

Although behavioral treatment research is limited for children with FXS [13, 30], several studies have shown that FCT interventions for problem behaviors can be successfully implemented via telehealth for young children with ASD [53, 54]. In a recently published randomized controlled trial [55], 38 children with ASD ages 1 to 7 years were randomized to receive either FCT delivered via telehealth (*n* = 21) or to continue treatment as usual (*n* = 17) over 12 weeks. Treatment as usual included ongoing psychoactive medications and other behavioral supports such as occupational therapy and/or speech therapy. A pre-treatment functional analysis (FA) showed that all participants exhibited problem behavior that was maintained by at least one social function (i.e., escape, attention, and/or access to tangibles). In-session observations of problem behavior indicated that children who received FCT via telehealth exhibited significantly greater decreases in problem behavior compared to those in the treatment as usual group (effect size = 1.57). Treatment acceptability was also found to be high at the end of FCT treatment.

Although these data provide strong support for the efficacy of FCT, it is unclear whether improvements in problem behavior occurred during times when the therapist was not available via telehealth. More general measures of problem behavior, for example, scores obtained on the Aberrant Behavior Checklist—Community (ABC-C) [56], could be employed in addition to in-session observations. Furthermore, it would be important to identify the different factors underlying treatment acceptability of FCT via telehealth. For example, did families consider FCT via telehealth to be a reasonable intervention, how willing were they to carry out the intervention, and how effective did they find FCT via telehealth to be? Finally, it would be important to determine whether the intervention had any impact on caregiver stress.

To improve the outcomes for children with FXS, and potentially reduce healthcare costs for the FXS community at large, we employed a randomized controlled design to compare children with FXS who received FCT via telehealth to those who received treatment as usual. In addition to in-session observations of problem behavior, we administered the ABC-C at 4-week intervals to determine overall changes in levels of problem behavior in each group. To examine the factors underlying treatment acceptability, we administered the Treatment Acceptability Rating Form—Revised (TARF-R) [57] at 4-week intervals for the FCT treatment group. Finally, to examine effects on parenting stress, caregivers of children in both groups completed the Parenting Stress Index, 4th edition [58] at baseline and at 12 weeks. We had three research questions: First, to what extent does implementing FCT via telehealth result in improvements in problem behavior in boys with FXS? Second, is FCT treatment via telehealth an acceptable intervention for caregivers of boys with FXS? Third, does FCT delivery via telehealth result in decreased levels of parental stress?

## Methods

To identify participants for the study, we advertised the study on social media (e.g., Facebook, Twitter) and sent emails with flyers and study information to caregivers of individuals with FXS via the National Fragile X Foundation. All advertising was pre-approved by the Stanford University IRB and invited parents to complete an online screening survey located on Research Electronic Data Capture (REDCap). The online survey included basic demographic questions concerning the child’s gender, age, and diagnosis, as well as questions from the Behavior Problems Inventory—Short Form (BPI-SF) [59] to determine the extent to which the child exhibited problem behavior. Children were included in the study if they were male, had a confirmed genetic diagnosis of FXS (> 200 CGG repeats on the *FMR1* gene with evidence of aberrant methylation), were aged between 3 and 10 years inclusive, and were reported to exhibit at least one form of problem behavior on a daily basis according to the BPI-SF. Families were also required to have Internet service at home with a signal that could support video-streaming capability. Children were excluded from the study if they had a significant sensory impairment (e.g., blindness or deafness), a neurological condition (e.g., frequent seizures, brain injury, Tourette’s syndrome), or if they received Applied Behavior Analysis (ABA) services in excess of 5 h per week. Finally, caregivers were asked to ensure that their child’s other therapies (i.e., medications or other treatments) remained as stable as possible throughout involvement in the study.

**Fig. 1:**
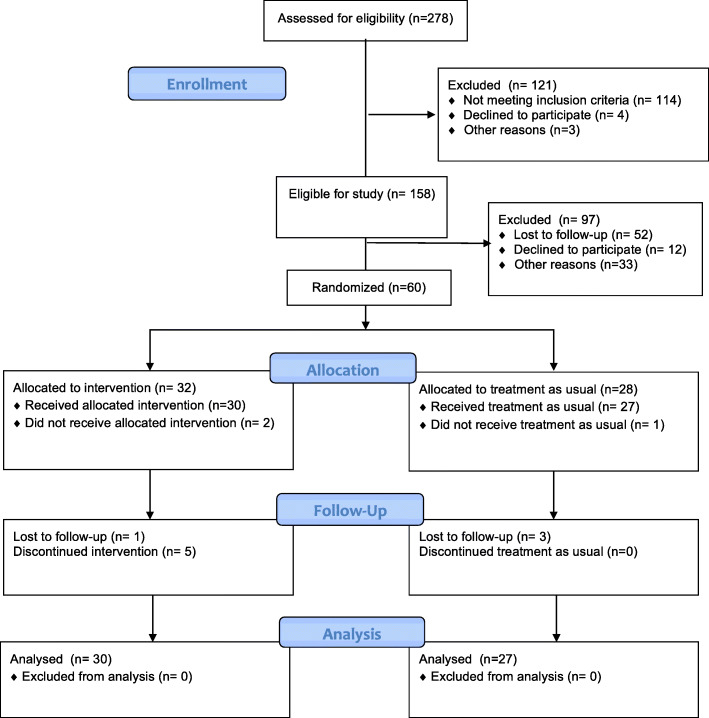
CONSORT diagram of subject flow through the study

We received responses from 278 families of children with FXS, 158 of whom met the initial study inclusion criteria. These families were subsequently contacted by telephone to obtain further information concerning their willingness to participate in the training, as well as any therapeutic services (e.g., speech and occupational therapy hours per week) and any other barriers to intervention. Figure 1 shows a CONSORT diagram of the subject flow through the project. Sixty families were initially enrolled in the project, and following collection of baseline data were randomized to receive either telehealth-enabled FCT or treatment as usual. Randomization was conducted in REDCap in permutated blocks of four using a table of values obtained from the online software tool available at randomization.com. Two families randomized to the treatment group withdrew before the intervention could be implemented due to scheduling concerns or changes in financial circumstances, and one family randomized to treatment as usual also subsequently withdrew due to unknown reasons. Thus, the analysis was conducted on 30 children who received telehealth-enabled FCT and 27 children who continued with treatment as usual. Note that data from the first 10 participants randomized to the treatment group are described in Monlux et al. [60].

Table 1 shows the demographic characteristics of the parent-child dyads randomized to each group. The mean age of the children was 6.8 years (SD = 2.4 years, range = 3.2 to 10.7 years), and the mean expressive communication age equivalent on the Vineland Adaptive Behavior Scales, 2nd Edition (VABS-II) [61] was 2.6 years (SD = 1.4 years, range = 0.3 to 7.5 years). The baseline scores obtained on the ABC-C were approximately 1 SD above the mean for similar aged children with intellectual disability [56]. The mean age of the primary caregivers was 39.0 years (SD = 7.6 years, range = 23.1 to 57.4 years). The majority of primary caregivers were married females with a college education, who lived in a suburban area and had a total household income of 100 K or less. There were no differences between the groups in child or parent characteristics.

**Table 1:** Demographic characteristics of parent-child dyads randomized to each group

	FCT via telehealth (*N*=30)	Treatment as usual (*N*= 27)
*Child characteristics*		
Age in years (*M*, SD)	6.64 (2.47)	7.04 (2.29)
Race/ethnicity		
Caucasian	80.0%	77.8%
Hispanic/Latino	16.7%	14.8%
African American	3.3%	0%
Pacific Islander	0%	7.4%
Adaptive behavior in years (M,SD)^a^		
Receptive communication age	2.36 (1.15)	3.06 (1.65)
Expressive communication age	2.34 (1.33)	2.94 (1.49)
Aberrant behavior (M, SD)^b^		
Irritability	19.8 (9.68)	17.85 (9.47)
Lethargy/social withdrawal	8.1 (7.35)	5.52 (6.94)
Stereotypic behavior	6.97 (5.05)	5.19 (3.70)
Hyperactivity/noncompliance	23.17 (11.18)	21.60 (8.66)
Inappropriate speech	3.67 (3.38)	3.81 (3.01)
Psychotropic medication use (%)	56.7%	70.4%
Autism diagnosis (%)	13.3%	25.9%
Epilepsy diagnosis (%)	13.3%	3.7%
*Parent Characteristics,*		
Age in years (M, SD)	39.44 (8.08)	38.47 (7.07)
Sex (F%)	96.7%	85.2%
Marital status		
Single	6.7%	11.1%
Married	93.3%	81.5%
Divorced	0%	7.4%
Household location		
Urban	16.7%	22.2%
Suburban	66.7%	63.0%
Rural	16.7%	14.8%
Household income		
Below 100 k	76.7%	70.4%
Above 100 k	23.3%	29.6%
Education level		
High school graduate	10.3%	22.2%
Partial college	31.0%	14.8%
College graduate	27.6%	37.0%
Graduate degree	17.2%	14.8%

^a^Vineland Adaptive Behavior Scales, 2nd edition [61]

^b^Aberrant Behavior Checklist—Community [56]

### Measures

The *Aberrant Behavior Checklist—Community* (ABC-C) [56] is a 58-item parent-report measure of problem behaviors commonly exhibited by children and adults with developmental disabilities. Items on the ABC-C are rated on a 4-point severity scale from 0 to 3 and resolve into five subscales: *irritability* (15 items), *lethargy/social withdrawal* (16 items), *stereotypic behavior* (7 items), *hyperactivity/noncompliance* (16 items), and *inappropriate speech* (4 items). Test-retest reliability coefficients for the 5 subscales of the ABC-C range from .80 to .95. The ABC-C was completed by caregivers at baseline, 4 weeks, 8 weeks, and 12 weeks to measure the severity of problem behavior.

The *Treatment Acceptability Rating Form—Revised* (TARF-R) [57] is a 21-item questionnaire designed to measure treatment acceptability for families who received behavioral treatment. Items are scored on a 7-point scale from 1 to 7 and are organized into six subscales: *reasonableness* (e.g., “Given your child’s behavioral problems, how reasonable do you find the treatment to be?”); *effectiveness* (e.g., “How confident are you that the treatment will be effective?”*)*; *willingness* (e.g., “How willing are you to carry out this treatment?”); *cost* (e.g., “How costly will it be to carry out this treatment?”); *side effects* (e.g., “To what extent are undesirable side-effects likely to result from this treatment?”); and *disruptiveness* (e.g., “How disruptive will it be to the family to carry out this treatment?”). Scores on each subscale range from 3 to 21. High scores on the *reasonableness*, *effectiveness*, and *willingness* subscales would indicate high treatment acceptability, whereas high scores on the *disruptiveness*, *cost*, and *side effects* subscales would indicate low treatment acceptability. The TARF-R has good internal consistency with Cronbach’s alpha coefficients ranging from .85 to .95 with a mean of .90. The TARF-R was completed at week 1, week 4, week 8, and week 12 by caregivers randomized to the treatment group.

The *Parenting Stress Index, Fourth Edition* (PSI-4) [58] is a 120-item inventory commonly employed to evaluate the parenting system and identify issues that may lead to problems in the child’s or parent’s behavior. The PSI-4 contains six subscales that measure sources of stress related to child characteristics (distractibility/hyperactivity, adaptability, reinforces parent, demandingness, mood, and acceptability) and seven subscales (competence, isolation, attachment, health, role restriction, depression, and spouse relationship) that measure sources of stress related to parent characteristics. These subscales are summarized by child domain, parent domain, and total stress scores. Test-retest reliability coefficients range from .55 to .82 for the child domain, from .69 to .91 for the parent domain, and from .65 to .96 for the total stress score. The PSI-4 was completed at baseline and at 12 weeks by caregivers in each group.

Prior to initiation of treatment, a member of the study team, who was a Board Certified Behavior Analyst (BCBA), traveled to the child’s home to conduct a functional analysis (FA) of the child’s problem behavior [60]. Briefly, six FA test conditions (i.e., *ignore*, *attention*, *tangible*, *social escape*, *academic escape*, and *transition escape*) and one control condition (*play*) were conducted at least 4 to 6 times over 2 days in the child’s home [33, 60]. In the *ignore* condition, the child was observed with no leisure items (e.g., toys) in the room, and caregivers were coached to ignore the child’s problem behavior. In the *attention* condition, the child was given access to a moderately preferred leisure item, and caregivers were coached to provide attention for 20 s if problem behavior occurred. In the *tangible* condition, caregivers were coached to remove a highly preferred leisure item but to give the item back for 20 s if problem behavior occurred. In the *social escape* condition, caregivers were coached to engage in social interaction with the child but to discontinue the interaction for 20 s if problem behavior occurred. In the *academic escape* condition, caregivers were coached to present academic demands but to remove these demands for 20 s if problem behavior occurred. In the *transition escape* condition, caregivers were coached to allow a transition between activities but to cancel the transition for 20 s if problem behavior occurred. Finally, in the *play* (control) condition, caregivers were coached to give the child access to attention and leisure items without presenting demands. Each condition was presented for 5 min followed by a 5-min break before the next condition was presented. Conditions were presented in the same order, i.e., ignore, attention, tangible, social escape, academic escape, transition escape, and play. During this same visit, the BCBA also determined the most appropriate room in the house to administer the treatment and assisted with setting up the iPad for Zoom. The BCBA then made a test call to another study team member (who was also a BCBA) via Zoom to ensure that the Internet connection was stable and the technology was otherwise working properly.

### Setting and technology

All treatment procedures were conducted in the child’s home by selecting an appropriate area of the house (e.g., dining room, living room, bedroom, or home office). The area was required to be at least 6′ × 6′, clear of any breakable objects, and to have controlled exits. Prior to beginning treatment, the BCBA provided guidance in-home to the caregiver concerning how to organize the materials and arrange the room (typically the same room that was used for the FA) to prevent the participant from leaving the range of the iPad camera. A text reminder was sent before each session to remind the caregiver to charge the iPad and earpiece and to provide a tentative agenda of the training goals for that day’s treatment session.

Our videoconferencing protocol was formulated to facilitate and troubleshoot any connection issues or problems establishing communication with the parent. All treatment sessions were conducted via Zoom which is approved by Stanford University for the transmission of “High Risk” data, including protected health information (PHI). To standardize the telehealth procedures across all study participants, each family received a technology kit that included an iPad tablet, as well as wireless earbuds for audio, a metal tablet stand, an iPad charger, laminated communication cards, and a step-by-step parent-friendly instruction booklet with checklists and technology troubleshooting information. All treatment sessions were video-recorded via Zoom using the built-in iPad camera.

Typically within 1 week of the in-home FA being completed, three remote meetings with the caregiver were scheduled. In the first meeting, both BCBA team members gave feedback concerning the results of the FA and proposed the FCT intervention over Zoom. After verbally approving the intervention, caregivers then participated in a second 60-min training session to establish rapport and provide training concerning the treatment protocol, safety strategies, and the parent’s role in the intervention’s delivery. During this session, the BCBAs used the screen-sharing feature of the video conferencing program to share PowerPoint slides and video models of the procedures. Finally, caregivers had an optional third meeting to run through each protocol once with their child and troubleshoot the technology and environmental setup. Caregivers were provided a laminated 12-page procedural manual with user-friendly flowcharts outlining the intervention procedures that would be implemented via telehealth, as well as the expected timeline for each step of the protocol. Caregivers were encouraged to review the written information before beginning the telehealth sessions as well as to call or text the study team if they had any questions about the procedures or any aspect of the study.

### Telehealth-enabled FCT

The treatment protocol was tailored to the results of each child’s FA [60]. For example, if the results of the FA indicated that the function of the child’s problem behavior was to gain access to attention and/or tangibles, the caregiver was coached to ignore the problem behavior when it occurred and to provide attention and/or preferred items for an appropriate alternative communicative response. If the function of the child’s problem behavior was determined to be escape from demands and/or transitions, the caregiver was coached to continue delivering task demands/transitions when the problem behavior occurred and to provide a break from the task for an alternative appropriate communicative response. For problem behavior maintained by more than one function (28 out of 30 participants), each function was targeted separately in 5-min blocks during each 1-h session using an alternating treatment design. Caregivers were also taught supplemental behavior analytic strategies (e.g., response blocking).

Based on best practice guidelines, the caregiver was initially taught to provide immediate and continuous reinforcement each time their child engaged in the appropriate communicative response to strengthen the new replacement communication. To prevent a loss in treatment effects when natural caregiver delays to providing reinforcement would inevitably occur, children were subsequently taught to tolerate delays by systematically increasing the response requirement or the amount of time required to wait for the reinforcer [60]. To increase generalization effects, we simulated environmental conditions that the child regularly encountered at home through collaboration with the caregivers.

Each 1-h treatment session began with a 10-min check-in with the family to review the agenda for the day, as well as a progress update on parent fidelity and the child’s treatment progress. The BCBA provided in vivo coaching and feedback to the caregivers as they implemented the relevant treatment procedures through a Bluetooth earpiece with their child. Coaching took the form of verbally prompting the caregiver to implement the intervention techniques, providing suggestions on activities, and giving feedback on the caregiver’s performance. Feedback involved praising the parent for implementing a particular procedure correctly (e.g., “Nice job ignoring the problem behavior”) or correcting procedural errors (e.g., “Please ignore the problem behavior, continue presenting the demand, and praise him for appropriate behavior”). Sessions were initially administered 5 days per week to build treatment momentum and then gradually faded by one session over successive weeks until sessions were being conducted once or twice per week, depending on progress.

All telehealth sessions were scheduled at a convenient time of day for the family (e.g., late afternoon or early evening), and each treatment session ended with a 10-min debriefing with the family to answer any questions. If problem behavior occurred at the end of a session, the study team member remained on videoconference until the behavior had de-escalated. To mitigate against potential injury during treatment or assessment, caregivers were offered safety equipment (e.g., arm guards, shin pads, knee pads, goggles) and informed about additional safety strategies (e.g., wearing hair up, removing earrings, ensuring that child’s fingernails were clipped) prior to the evaluation. We also established session termination criteria and an emergency response protocol to ensure child and caregiver safety. Specifically, if the child engaged in an extremely high level of problem behavior that would result in injury to the child or others (e.g., bruising, bleeding) or significant damage to property, the session would be immediately terminated so that appropriate first aid could be administered.

### Caregiver fidelity of implementation

A protocol outlining each step in the treatment was used to score caregiver fidelity with the intervention. Caregivers received a correct score for a given intervention strategy if they correctly implemented the strategy as described in the protocol independently. Incorrect responses were classified as errors of commission (i.e., incorrect implementation of a step) or errors of omission (i.e., failure to perform a step), and an incorrect score was recorded if the caregiver implemented the strategy incorrectly or received guidance from the clinician. Treatment fidelity for the intervention was measured for approximately 40% of sessions across participants. The mean percentage fidelity was 95.7%, and the range was 81.5 to 99.3% across participants.

### Treatment as usual

Participants randomized to treatment as usual did not receive the intervention, but caregivers were allowed to continue any other treatments with their child as they typically would over 12 weeks (as long as ABA remained less than 5 h per week). Treatment as usual included any medications or additional therapies that their child was receiving, such as speech therapy or occupational therapy. If there were any unanticipated changes in medications or additional therapies, these changes were noted.

### In-session observations

Observations of problem behavior were conducted while the caregiver implemented the intervention procedures directly with their child in each session by trained raters who were BCBAs. Given that observations were conducted only for children who received treatment, the coders were not naïve to the treatment condition. *Problem behavior* was defined as any behavior that could potentially result in tissue damage to the caregiver (e.g., hitting, kicking, throwing items at the caregiver), the child’s own body (e.g., self-biting, self-hitting), and/or any behavior that could potentially result in damage to property (e.g., kicking items, throwing items, ripping books). A second observer independently scored 30 to 40% of the video data for each participant to establish the inter-rater reliability of the recorded frequencies of problem behavior. Intraclass correlation coefficients (ICC) were above 0.95 for each participant.

### Data analysis

To determine the function(s) of each child’s problem behavior, data from each FA was examined using a combination of visual analysis and our recently published method, automated nonparametric statistical analysis (ANSA) [62]. Briefly, ANSA employs nonparametric statistical tests to determine whether the rate of problem behavior observed in a test condition (e.g., attention) is differentiated from the play (control) condition and/or if there is a significant upward or downward trend in a test condition. Functions for problem behavior were identified by determining if the rate of problem behavior observed in a test condition was significantly higher than the rate of problem behavior in the play condition and/or if there was a significant upward trend in the rate of problem behavior observed within a test condition. Functions for problem behavior were identified as attention, tangible, social escape, academic escape, and/or transition escape.

Our primary outcome measure was the score obtained on the irritability subscale of the ABC-C. The irritability subscale of the ABC-C contains items relating to aggression, self-injury, tantrums, agitation, and unstable mood and is commonly employed as an outcome measure in clinical trials. Secondary outcome measures were scores obtained on the other ABC-C subscales and the PSI-4. We employed standard linear mixed-effects modeling [63, 64] to estimate changes from baseline to end of treatment in our primary and secondary outcomes. In line with the intention-to-treat (ITT) principle, we included all randomized individuals in the analyses as long as their data were available from at least one of the assessments. For all model estimation, we used the maximum likelihood embedded in the Mplus program [65]. Specifically, we estimated linear change over time, allowing for random intercepts and slopes. Effect sizes (Cohen’s *d*) were calculated based on the observed standard deviation pooled across the treatment and control conditions at the end of treatment. Data points that were missing due to attrition or missing assessments were handled, assuming that data were missing at random conditional on observed information [66]. To provide an in-session metric of problem behavior, we calculated the rate of problem behavior observed in each session and averaged these rates for each week of treatment across each child. Rates of problem behavior during the last week of treatment were then compared to the baseline rate observed in the child’s FA.

## Results

### Baseline functional analyses

For children in the treatment group, social functions for problem behavior were identified for 28 (93.3%) participants, with 8 (26.7%) boys displaying problem behavior maintained by a single function and 20 (67.7%) boys displaying problem behavior maintained by multiple social functions. The most common combination of functions identified was tangible reinforcement and escape from demands and/or transition escape.

Overall, 24 of the 30 families enrolled in the treatment group completed the FCT treatment. Three families withdrew after 1 week of treatment due to scheduling concerns, one family withdrew after 2 weeks due to illness-related issues, one family withdrew after 4 weeks of treatment because the caregiver was finding it difficult to implement the treatment when problem behavior occurred, and one family withdrew after 6 weeks of treatment due to work conflicts. Three families were lost to follow-up in the treatment as usual group. Overall, the median number of telehealth sessions that were completed as scheduled across participants in the treatment group was 37 (range = 2 to 47), whereas the median number of telehealth sessions that needed to be rescheduled was 1 (range = 0 to 5). The median number of treatment sessions that were interrupted due to technology issues was 0 (range = 0 to 2).

### Problem behavior

The results of the intention-to-treat analyses based on mixed-effects modeling for the outcomes on the ABC-C are summarized in Table 2.

**Table 2:** Estimated intention-to-treat effects on changes in problem behavior on the ABC-C subscales based on mixed effects modeling

	Baseline to end of treatment (12 weeks)		
ABC-C Subscale	FCT via telehealth	Treatment as usual	Group difference
Irritability	− 6.44 (*p* < .001)	− 1.35 (*p* = .263)	− 5.09 (*p* < .001, *d* = .65)
Lethargy/social withdrawal	− 2.32 (*p* = .007)	− .89 (*p* = .259)	− 1.43 (*p* = .149, *d* = .19)
Stereotypic behavior	− 2.17 (*p* < .001)	− .94 (*p* = .080)	− 1.22 (*p* = .042, *d* = .34)
Hyperactivity/noncompliance	− 6.66 (*p* < .001)	− 1.40 (*p* = .212)	− 5.25 (*p* = .005, *d* = .58)
Inappropriate speech	− .65 (*p* = .05)	.11 (*p* = .778)	− .76 (*p* = .145, *d* = .22)

In all analyses, we focused on the change from baseline to end of treatment at 12 weeks. For the treatment group, the mean score obtained on the irritability subscale of the ABC-C decreased from baseline by 6.44 points at 12 weeks, a 42.6% decrease on average. Conversely, the mean score on the irritability subscale of the ABC-C for the treatment as usual group decreased from baseline by 1.35 points at 12 weeks, a 9.13% decrease on average. A comparison of the groups indicated that the FCT group showed significantly greater improvement compared to treatment as usual (*p* < .001, Cohen’s *d* = 0.65). The rate of a positive response (at least a 25% improvement in score on the irritability subscale of the ABC-C at 12 weeks) was 66.7% (16 of 24) for children in the FCT treatment group and 29.2% (7 of 24) for children in the treatment as usual group (*χ*^2^(1) = 6.76, *p* = .009). The FCT group also showed significantly greater improvements on the stereotypic behavior subscale of the ABC-C (*p* = .042, *d* = 0.34) as well as on the hyperactivity/noncompliance subscale of the ABC-C (*p* = .005, *d* = 0.58) compared to the treatment as usual group.

**Fig. 2:**
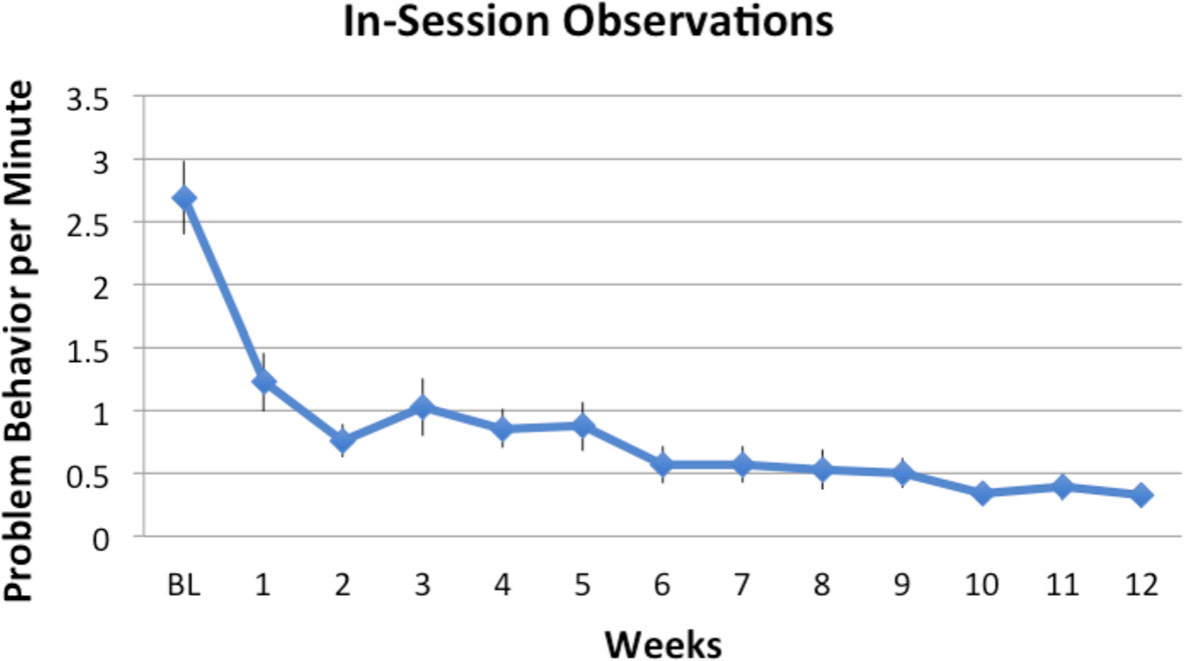
In-session rates of problem behavior observed at baseline (BL) and during each week of FCT treatment. Error bars are standard errors

Figure 2 shows the rates of problem behavior plotted for the FA baseline and during each week of treatment for boys who received FCT treatment. The mean rate of problem behavior observed at baseline was 2.70 responses per minute (*SD* = 1.57 rpm). Mean levels of problem behavior decreased during the first week of FCT treatment to approx. 1.25 rpm and remained at 1 rpm until the fifth week of treatment. Mean levels of problem behavior subsequently decreased to 0.5 rpm during weeks 6 to 9 and remained just below 0.5 rpm by the end of treatment.

Overall, there was a steady decrease of in-session rates of problem behavior from baseline to end of treatment with the average percentage decrease being 91.7% (range = 74.2 to 100%). To examine whether parent fidelity was associated with child behavior change, we correlated the percentage fidelity rate with the percentage decrease in problem behavior. There was a significant positive association (*r* = .59, *p* = .003) indicating that increased fidelity of implementation resulted in greater reductions in problem behavior.

### Treatment acceptability

**Fig. 3:**
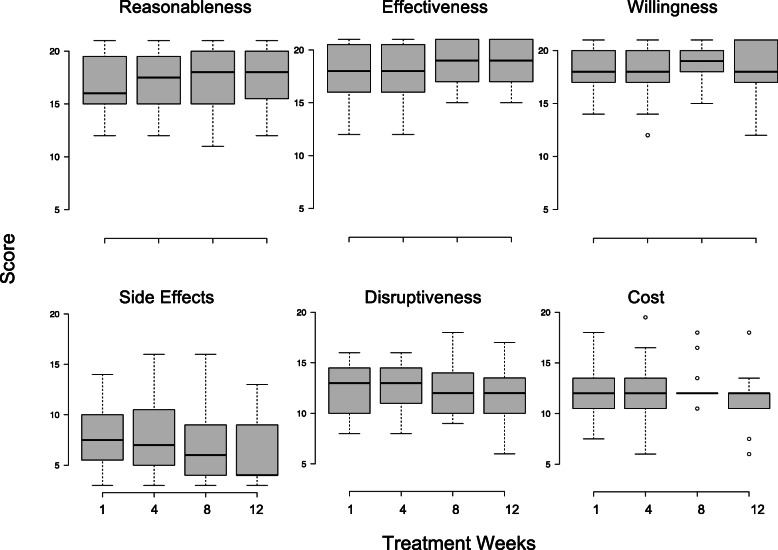
Distribution of scores obtained on each subscale of the Treatment Acceptability Rating Form—Revised (TARF-R) at 4-week intervals across treatment

Figure 3 shows the treatment acceptability data obtained on the TARF-R measured at weeks 1, 4, 8, and 12 for children randomized to the treatment group.

At baseline, high scores were obtained on the *reasonableness* (*M =* 17.9, SD *=* 2.5), *effectiveness* (*M =* 16.9, SD *=* 2.8), and *willingness* (*M =* 18.4, SD *=* 1.9) subscales of the TARF-R, indicating that the FCT procedures were rated to be highly reasonable and effective and that caregivers were also very willing to carry out FCT with their child. The corresponding scores on the *cost* (*M =* 12.4, SD *= 2.6*) and *disruptiveness* (*M =* 12.4, SD *=* 2.4) subscales were in the middle range, indicating that the FCT procedures were rated as neither costly nor disruptive on the family. The scores on the *side effects* (*M =* 7.8, SD *=* 2.9) subscale were in the low range, indicating that side effects were minimal. The scores on each subscale of the TARF-R remained stable across treatment weeks indicating that treatment acceptability remained consistently high across treatment.

### Parenting stress

Table 3 shows the scores obtained on each subscale of the PSI-4 at baseline, and Table 4 shows the changes in parenting stress scores from baseline to 12 weeks for caregivers in each group.

**Table 3:** Mean T scores obtained on the Parenting Stress Index, 4th edition for each group at baseline

PSI-4 Subscale T score (*M*, SD)	FCT via telehealth	Treatment as usual
Child characteristics		
Distractibility/hyperactivity	68.3 (8.1)	70.1 (7.5)
Adaptability	62.3 (8.6)	62.4 (9.4)
Reinforces parent	50.2 (12.0)	49.1 (10.2)
Demandingness	67.2 (8.5)	64.5 (10.3)
Mood	60.4 (10.2)	57.8 (10.5)
Acceptability	62.6 (7.5)	61.5 (6.3)
Parent characteristics		
Competence	53.3 (8.8)	55.6 (9.2)
Isolation	55.1 (10.4)	58.2 (13.2)
Attachment	48.5 (8.4)	48.5 (7.9)
Health	54.3 (10.0)	55.9 (10.6)
Role restriction	56.5 (14.1)	56.9 (13.4)
Depression	53.1 (8.6)	53.8 (9.0)
Spouse relationship	50.8 (8.9)	53.5 (11.3)
Child domain	64.6 (7.0)	63.7 (7.3)
Parent domain	53.5 (8.3)	55.3 (9.2)
Total stress	59.0 (7.4)	59.7 (8.1)

**Table 4:** Estimated intention-to-treat effects on changes in parenting stress based on mixed-effects modeling

PSI-4 subscale	Baseline to end of treatment (12 weeks)		
	FCT via telehealth	Treatment as usual	Group difference
Child characteristics			
Distractibility/hyperactivity	− 4.037 (*p* = 0.004)	0.705 (*p* = 0.650)	− 4.742 (*p* = 0.024, *d* = 0.47)
Adaptability	− 1.925 (*p* = 0.182)	− 0.417 (*p* = 0.660)	− 1.508 (*p* = 0.370, *d* = 0.15)
Reinforces parent	− 4.665 (*p* = 0.014)	0.197 (*p* = 0.913)	− 4.862 (*p* = 0.068, *d* = 0.54)
Demandingness	− 4.989 (*p* = 0.007)	− 1.278 (*p* = 0.496)	− 3.712 (*p* = 0.161, *d* = 0.38)
Mood	− 5.392 (*p* = 0.000)	2.779 (*p* = 0.054)	− 8.170 (*p* = 0.000, *d* = 0.83)
Acceptability	− 1.476 (*p* = 0.262)	− 0.924 (*p* = 0.497)	− 0.552 (*p* = 0.770, *d* = 0.08)
Parent characterisitcs			
Competence	− 2.997 (*p* = 0.037)	− 4.871 (*p* = 0.001)	1.875 (*p* = 0.373, *d* = 0.19)
Isolation	− 2.263 (*p* = 0.231)	− 1.148 (*p* = 0.504)	− 1.115 (*p* = 0.664, *d* = 0.09)
Attachment	− 0.967 (*p* = 0.638)	− 0.490 (*p* = 0.763)	− 0.477 (*p* = 0.862, *d* = 0.06)
Health	− 2.510 (*p* = 0.151)	− 1.237 (*p* = 0.512)	− 1.273 (*p* = 0.627, *d* = 0.11)
Role restriction	− 2.670 (*p* = 0.120)	0.727 (*p* = 0.551)	− 3.396 (*p* = 0.108, *d* = 0.29)
Depression	− 2.745 (*p* = 0.137)	1.661 (*p* = 0.245)	− 4.407 (*p* = 0.059, *d* = 0.42)
Spouse relationship	− 1.364 (*p* = 0.326)	0.212 (*p* = 0.860)	− 1.577 (*p* = 0.402, *d* = 0.16)
Child domain	− 4.321 (*p* = 0.001)	− 0.326 (*p* = 0.748)	− 3.995 (*p* = 0.013, *d* = 0.49)
Parent domain	− 2.414 (*p* = 0.097)	− 1.062 (*p* = 0.296)	− 1.352 (*p* = 0.453, *d* = 0.14)
Total stress	− 3.362 (*p* = 0.010)	− 0.734 (*p* = 0.318)	− 2.628 (*p* = 0.083, *d* = 0.31)

At baseline, the highest sources of parental stress were related to characteristics of the child, including *distractibility/hyperactivity* (*M* = 69.1, *SD* = 7.8) and *demandingness* (*M* = 65.9, *SD* = 9.4). For caregivers in the FCT treatment group, significant improvements were obtained on the *distractibility/hyperactivity* (*p* = .004), *reinforces parent* (*p* = .014), *demandingness* (*p* = .007), and *mood* (*p* < .001) subscales following treatment. There were significant improvements on the *child domain* (*p* = .001) and *total stress* (*p* = .010) scales in this group following treatment. There were also significant improvements on the *competence* subscale for boys who received FCT treatment (*p* = .037) as well as for boys who received treatment as usual (*p* = .001). Comparison of the groups indicated that caregivers in the FCT treatment group showed significantly greater improvements on the *distractibility/hyperactivity* (*p* = .024, *d* = .47) and *mood* subscales (*p* < .001, *d* = .83), as well as on the *child domain* (*p* = .013, d = .49) of the PSI-4 compared to the treatment as usual group.

## Discussion

The status quo as it pertains to the treatment of problem behavior for children with FXS is currently limited to pharmacological interventions and/or behavioral strategies that do not take the potential function(s) of problem behavior into account. Furthermore, when behavioral treatments are administered for FXS, they are traditionally implemented in clinic-based models of care. Given that the majority of clinics that specialize in FXS are located in larger cities, families affected by FXS, who are based in rural communities, may not be able to access clinical care. This study represents a substantive departure from the status quo by administering a targeted behavioral approach, based on behavior analytic principles, for treating problem behavior using a telehealth-based delivery model.

In this longitudinal RCT, the ABC-C was administered at 4-week intervals, allowing for strong inference regarding the longitudinal relationships among behavioral treatment and problem behaviors in children with FXS. Our data indicated that problem behavior decreased significantly on the irritability subscale of the ABC-C, our primary outcome measure. There were also significant decreases in the stereotypic behavior and hyperactivity/noncompliance subscales of the ABC-C. Further, direct observational data collected during each session showed that in-session levels of problem behavior had decreased substantially by 91% on average at the end of treatment compared to baseline. Importantly, the data showed that parent treatment fidelity was associated with greater reductions in problem behavior, indicating that increased treatment fidelity was associated with better child outcomes. Taken together, these data indicate that FCT delivered via telehealth can result in significant reductions in problem behavior commonly displayed by children with FXS.

We employed the irritability subscale of the ABC-C as the primary outcome measure in this study because this metric is often employed in studies evaluating the efficacy of medications for children with developmental disabilities, including children with FXS [67–70]. However, given that caregivers were not naïve to the study procedures, the ABC-C ratings could have been biased. Although the ABC-C is a general measure of problem behavior, the use of a tool or outcome measure more distal from the treatment could also have been employed to bolster the findings. The lack of generalizable measures is a challenge for all telehealth-mediated treatments in which participants do not travel to the lab or clinic for evaluations with naïve assessors. Behavior ratings by naïve raters unaware of group membership would address one aspect of this (although the ratings would still be contextually bound to home and treatment sessions). In the present study, the effect size for the difference between the FCT group and the treatment as usual group on the irritability subscale of the ABC-C at the 12-week outcome point was 0.65, a similar effect size to that reported in clinical trials of risperidone for the treatment of problem behaviors shown by children with ASD [67, 68]*.* The mean score on the irritability subscale of the ABC-C at baseline for the current sample was 18 points, approximately 1 standard deviation above the population mean for samples of male children with developmental disabilities in the same age range [71] and a decrease of 6.44 points on this subscale is slightly less than 1 SD. Future studies will be needed to compare the efficacy between behavioral treatment with FCT and pharmacological treatment in FXS. Ultimately, it is possible that a combination of pharmacological and behavioral treatment may be the most beneficial treatment strategy [13, 72].

Direct parent involvement in behavioral treatment is a crucial component in achieving long-term success for FCT outcomes because parents are responsible for the provision of care, supervision, and managing their child’s behavior during all hours outside of their child’s school program [73]. Long-term intervention success has been found when those who interact with the child on a daily basis (i.e., primary caregivers) are involved at every stage of the treatment process [74–79]. Parent-mediated interventions have been effective in training parents to implement a variety of behavioral strategies with children with ASD, yet have been relatively unused as a clinical treatment model with FXS. Parents who care for a child with a disability experience a high level of stress and increased mental and physical health concerns, and this stress leads to the increased undue burden placed on the family, resulting in poor child outcomes and quality of life [80–82]. In the present study, we found that the main sources of stress for caregivers were related to characteristics of the child, specifically distractibility/hyperactivity and mood. These elements of stress were more likely to decrease for caregivers whose children received FCT treatment compared to those who received treatment as usual. However, the treatment appeared to have limited impact on parental sources of stress although improvements concerning the extent to which the parent felt competent in the parenting role were obtained in both groups. Given the impact of these child stressors, it is imperative that researchers and clinicians gain a better understanding of how these family factors relate to treatment outcomes. These factors will provide much-needed knowledge that will lead to improved clinical practices as it relates to the assessment of the child and family unit as a whole as well as the development of comprehensive treatment plans by a multidisciplinary team.

We attempted to minimize any technology challenges by developing a protocol to troubleshoot potential technology issues and to identify solutions to ensure the successful completion of the study. Troubleshooting protocols included assessment of Internet speed, minimizing the use of video streaming by other family members during sessions to improve bandwidth and using a Wi-Fi signal extender if needed. The clinician was able to call the caregiver if the Internet connection was lost at any time. In the event of technology failure, we maintained backup technology in our office so that we could overnight technology to families to minimize treatment delays. We note that our attrition rate (20%) is significantly lower than those commonly reported for other caregiver-mediated behavioral treatments [83]. It is possible that the willingness of caregivers to carry out the FCT treatment may be increased because the treatment is specifically matched to the function(s) of the child’s problem behavior, and thus the rationale for the treatment is clear. We recognize, however, that the clinician was not able to assist with the physical management of the problem behavior directly in the home, which carries risks to safety and attrition. Despite high levels of problem behavior, we were able to structure the treatment sessions to reduce risks of injury and therefore did not to need to terminate sessions for any of our participants.

These promising data collectively provide support for telehealth-enabled FCT as an effective intervention to reduce problem behavior in children with FXS aged 3 to 10 years. While our current study offers the potential to demonstrate the benefits of telehealth-enabled FCT as compared to treatment as usual (which is a clinically relevant and important comparison), it would be important to compare our intervention to a more active control condition in which caregivers are required to engage in a similar number of hours learning about behavioral issues and how to manage them. This would ensure that therapeutic time and input was similar across groups [78]. It would also be important to determine whether FCT delivered via telehealth may be more effective than FCT delivered “in-person” in a clinic or home. Another important consideration concerns the extent to which treatment gains are maintained, once direct behavioral coaching has ceased.

## Conclusion

Our study provides critical research support for the use of telehealth as a service delivery model for behavior analytic treatments in FXS. The selection of the most efficacious treatment for addressing problem behaviors in children with FXS is pivotal to increasing the long-term health and well-being of families. It is hoped that this approach will significantly impact the development of interventions for problem behaviors in all children with FXS in the future as well as those with other neurodevelopmental disorders. Employing telehealth may also offer a feasible and cost-effective approach to evaluating and addressing significant behavioral issues prior to considering pharmacological treatments.

## Data Availability

The data that support the findings of this study are not available due to them containing information that could compromise research participant consent.

## Abbreviations

FXS: Fragile X syndrome
ASD: Autism spectrum disorder
ABA: Applied behavior analysis
FA: Functional analysis
FCT: Functional communication training
RCT: Randomized controlled trial
BPI-SF: Behavior Problems Inventory—Short Form
ABC-C: Aberrant Behavior Checklist-Community
TARF-R: Treatment Acceptability Rating Form-Revised
PSI-4: Parenting Stress Index, 4th edition
BCBA: Board Certified Behavior Analyst
ANSA: Automated Nonparametric Statistical Analysis
